# Predictors of virological failure among people living with HIV receiving first line antiretroviral treatment in Myanmar: retrospective cohort analysis

**DOI:** 10.1186/s12981-021-00336-0

**Published:** 2021-04-21

**Authors:** Anita Mesic, Alexander Spina, Htay Thet Mar, Phone Thit, Tom Decroo, Annick Lenglet, Moe Pyae Thandar, Thin Thin Thwe, Aung Aung Kyaw, Tobias Homan, Mitchell Sangma, Ronald Kremer, Jane Grieg, Erwan Piriou, Koert Ritmeijer, Josefien Van Olmen, Lutgarde Lynen, Htun Nyunt Oo

**Affiliations:** 1 Public Health Department, Médecins Sans Frontières, Plantage Middenlaan 14, 1001DD Amsterdam, The Netherlands; 2 University of Exeter Medical School, Heavitree Road, Exeter, EX1 2LU UK; 3 Médecins Sans Frontières, Thingangyun Township, No 5/59, Ayeyadanar Street, Thirigon Villa, Waizayandar Road, Yangon, Myanmar; 4 Institute of Tropical Medicine, Department of Clinical Sciences, Kronenburgstraat 43, 2000 Antwerpen, Belgium; 5 Research Foundation Flanders, Egmontstraat 5, 1000 Brussels, Belgium; 6 Department of Medical Microbiology, Radboud University Medical Center, Geert Grooteplein Zuid 10, 6525 GA Nijmegen, The Netherlands; 7 Burnet Institute, 85 Commercial Road, Melbourne, VIC 300 Australia; 8 Department of Family Medicine and Population Health, University of Antwerp, Doornstraat 331, 2610 Antwerpen, Belgium; 9 Disease Control Office, National AIDS Programme, Naypyidaw, Myanmar

**Keywords:** HIV, First-line antiretroviral treatment, Virological failure, Lost-to-follow up, Low viremia, Myanmar

## Abstract

**Background:**

Progress toward the global target for 95% virological suppression among those on antiretroviral treatment (ART) is still suboptimal. We describe the viral load (VL) cascade, the incidence of virological failure and associated risk factors among people living with HIV receiving first-line ART in an HIV cohort in Myanmar treated by the Médecins Sans Frontières in collaboration with the Ministry of Health and Sports Myanmar.

**Methods:**

We conducted a retrospective cohort study, including adult patients with at least one HIV viral load test result and having received of at least 6 months’ standard first-line ART. The incidence rate of virological failure (HIV viral load ≥ 1000 copies/mL) was calculated. Multivariable Cox’s regression was performed to identify risk factors for virological failure.

**Results:**

We included 25,260 patients with a median age of 33.1 years (interquartile range, IQR 28.0–39.1) and a median observation time of 5.4 years (IQR 3.7–7.9). Virological failure was documented in 3,579 (14.2%) participants, resulting in an overall incidence rate for failure of 2.5 per 100 person-years of follow-up. Among those who had a follow-up viral load result, 1,258 (57.1%) had confirmed virological failure, of which 836 (66.5%) were switched to second-line treatment. An increased hazard for failure was associated with age ≤ 19 years (adjusted hazard ratio, aHR 1.51; 95% confidence intervals, CI 1.20–1.89; *p* < 0.001), baseline tuberculosis (aHR 1.39; 95% CI 1.14–1.49; *p* < 0.001), a history of low-level viremia (aHR 1.60; 95% CI 1.42–1.81; *p* < 0.001), or a history of loss-to-follow-up (aHR 1.24; 95% CI 1.41–1.52; *p* = 0.041) and being on the same regimen (aHR 1.37; 95% CI 1.07–1.76; *p* < 0.001). Cumulative appointment delay was not significantly associated with failure after controlling for covariates.

**Conclusions:**

VL monitoring is an important tool to improve programme outcomes, however limited coverage of VL testing and acting on test results hampers its full potential. In our cohort children and adolescents, PLHIV with history of loss-to-follow-up or those with low-viremia are at the highest risk of virological failure and might require more frequent virological monitoring than is currently recommended.

**Supplementary Information:**

The online version contains supplementary material available at 10.1186/s12981-021-00336-0.

## Background

There is a global commitment to end the AIDS epidemic by 2030 [1] and the global HIV response has improved access to care and survival among people living with HIV (PLHIV) [2]. However, by the end of 2018, virological suppression for PLHIV on antiretroviral treatment (ART) was 85%, which is still below the UNAIDS target of 95%. Scale-up of routine HIV viral load (VL) testing in resource-limited settings has been suboptimal due to the cost and complexity of VL testing, but also due to the lack of awareness about the benefits of regular VL monitoring among health care providers and patients [3]. In 2018, UNAIDS reported 49% (95% CI 38–63%) estimated rates of virological suppression among all PLHIV in the Asia and Pacific region [2].

In 2017, less than 5% of those globally receiving ART were thought to be receiving second-line ART [4]. A study from sub-Saharan Africa identified poor access to HIV VL monitoring as the main reason for a delayed switch to second-line treatment. VL monitoring was poorly used even when available in this cohort: 40% of patients with virological failure were not switched to second-line ART, whereas 30% had been switched without proof of failure [4]. A study from Myanmar reported high rates of virological failure, but low rates of switching to second-line treatment [5]. Lack of switching was attributed to clinical or programmatic factors, such as delayed reporting of the VL results, concerns about adherence or pill burden, or centralized decision making [6]. The cost of second-line treatment, was also prohibitive, being 2.5 times more expensive than the first-line therapy at the time [7]. Improper management of patients with treatment failure leads to poor treatment outcomes, accumulation and transmission of HIV drug resistance and increases cost of HIV care delivery [8, 9].

Myanmar has the second highest HIV prevalence in Southeast Asia with an estimated 0.57% of the general population being HIV-positive [2]. In 2018 there were an estimated 240,000 PLHIV in the country with the highest HIV burden among sex workers, men having sex with men and people who inject drugs [10]. The National AIDS Programme achieved 77% ART coverage by the end of 2019 [11]. Despite significant improvements in access to HIV care and national guidelines recommending routine HIV VL testing [12], only 72% of PLHIV on ART had access to VL monitoring in 2019 in the country [11]. Virological suppression among those who had access to HIV VL testing was 95%, thus it is on track to the 95% UNAIDS target [13].

Previous studies identified poor adherence [14–16], advanced HIV disease [15–18], tuberculosis co-infection [14], and longer time on first-line ART as predictors of ART failure [16]. Recent studies reported an association between having low-level viremia and virological failure [19, 20]. With the increasing life span of the HIV cohorts, it is increasingly common for people to interrupt treatment for a short period of time or to be lost-to-follow up (LFU) and then re-engage in care. Studies report that 11–77% of patients enrolled in HIV care temporarily disengage [21–25]. In most HIV programmes the frequency of treatment interruptions is very likely underestimated by most HIV programmes. HIV care is more complex for patients previously exposed to ART and at risk of HIV drug resistance, especially if presenting back into care with advanced HIV disease [26, 27]. The correlation between cumulative appointment delay and treatment failure has not been explored in any previous studies, to our knowledge.

Since 2003 Médecins Sans Frontières (MSF) has been providing HIV care in Yangon, Kachin and Shan States. VL monitoring was introduced in 2009, initially as a targeted approach for those most at risk of failure. Since 2016 all patients were eligible for routine HIV VL monitoring once per year. In this study we describe the VL cascade, the incidence of virological failure and associated risk factors, including the cumulative appointment delay, among PLHIV receiving first-line ART in the HIV cohort treated by MSF in Myanmar.

## Methods

### Design and study population

**Fig. 1:**
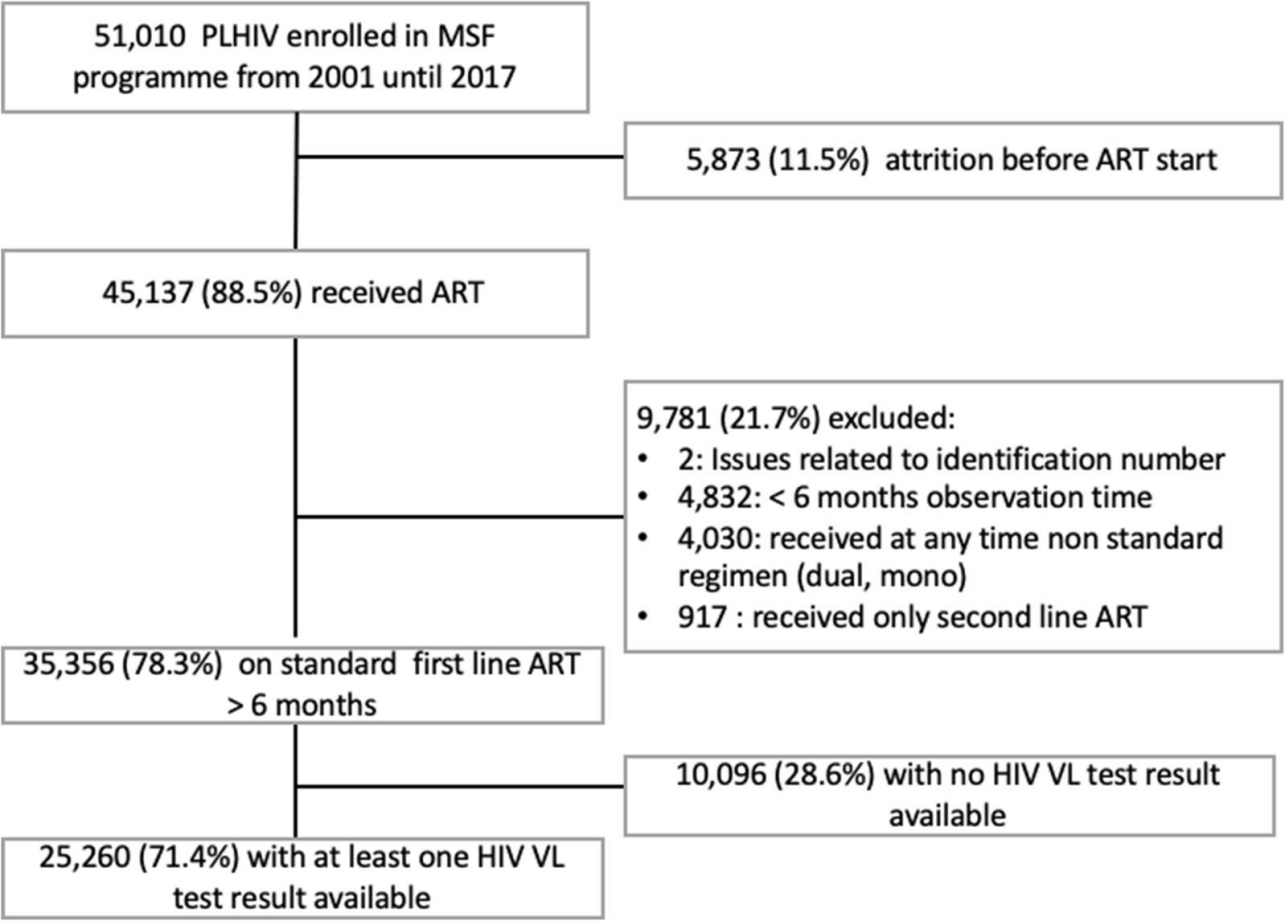
Flowchart of inclusion pathway in the study

We conducted a retrospective cohort study of patients enrolled on ART in the MSF HIV programme in Myanmar between 01 January 2001 and 31 October 2017. The study included patients who had at least one HIV VL test available after receiving at least 6 months of standard first-line ART (Fig. 1).

### Study setting

The study was conducted in the MSF HIV programmes in Yangon, Kachin and Shan States in Myanmar. The study sites provided a comprehensive package of HIV care free of charge. Clinical care was provided by medical doctors and nurses, while trained counselors and outreach adherence supporters provided counselling and adherence support [28]. Since 2009 HIV VL testing targeted those with immunological and/or clinical failure, and those switching their first-line regimen because of modified guidelines. From 2014 onwards, yearly routine VL monitoring targeted all children and patients on second-line ART. Since 2016, yearly routine VL testing was introduced for all patients on ART. Patients with viremia (VL > 200 copies/mL defined as a limit of detection) received 3–6 counselling sessions over a period of 3 months and were then prescribed a follow-up VL test. Those with two consecutive VL results above the threshold for failure (≥ 5000 copies/mL until 2012,  ≥ 1000 copies/mL after 2012) were started on a second-line ART regimen. If the follow-up VL did not show failure, enhanced adherence support continued together with three-monthly VL monitoring until undetectable viremia was observed.

For the first-line treatment a combination of two nucleoside/nucleotide reverse transcriptase inhibitors (zidovudine, tenofovir, lamivudine, abacavir) with a non-nucleoside reverse transcriptase inhibitor (efavirenz or nevirapine) was used. Second-line regimen was composed of two nucleoside/nucleotide reverse transcriptase inhibitors, ideally not used in the first-line regimen and one protease inhibitor (atazanavir/ritonavir or lopinavir/ritonavir).

### Study variables

The study used routine programme data collected from standardized patient forms and encoded in the MSF HIV programme database, FUCHIA (Follow-up and Care of HIV Infection and AIDS). Values recorded during the ART initiation visit were considered baseline for: age, marital status, gender, World Health Organization (WHO) stage, body mass index (BMI), risk group, and tuberculosis co-infection. We defined CD4 at ART initiation as the measurement taken closest to the date of ART initiation, within 92 days before or after initiation. BMI was used as a binary variable (< 18.5 kg/m^2^,  ≥ 18 kg/m^2^) and values > 40 kg/m^2^ were considered errors and defined as missing. Yearly coverage of viral load was defined as the proportion of patients active and in care at the end of the year, who had at least one VL measurement in that year. Virological failure was defined as a patient with a VL ≥ 1000 copies at a visit ≥ 183 days after ART initiation. Those with a follow-up VL ≥ 1000 copies/ml, within 183 days of a previous VL showing virological failure, had confirmed virological failure. Low-level viremia was defined as a VL between 200 and 999 copies, occurring ≥ 183 days after ART initiation, and prior to a first episode of virological failure. Starting dates were defined for each patient based on the earliest visit date at which ART was prescribed. If this date was before 2009, then the start date was set to 1st January 2009 for regression analyses. The reason for this is that VL testing only started to become widely available after 2009; thus, the need to avoid overweighting those who started treatment before 2009 but had no chance of having VL tests. For calculation of operational indicators in the cascade analysis, the original ART start date was used, regardless of whether this was before 2009 or not. End dates were defined based on the earliest occurring visit date at which there was either a virological failure, death, switch to second line ART or reached the maximum visit for that patient without event. For the definition of LFU, each visit had an expected next visit date. We calculated the difference between expected and actual next visit date in days. If this difference was greater than 60 days then the earlier visit was marked as LFU. Using this, the following variables were created: number of times a patient was LFU, total days patient was LFU (including the initial 60 days). Cumulative appointment delay was calculated as the number of days of delay between the dates of appointment and the actual dates visits took place. Time under observation was calculated as the time between starting and ending dates in years. Time on ART was calculated as time under observation minus time LFU.

### Data analysis

Baseline characteristics were described using frequencies and percentages for categorical variables and medians with interquartile ranges (IQRs) for continuous variables. We compared proportions for categorical variables using a chi-squared test (with Holm correction) and differences in distribution for continuous variables using a Kruskal–Wallis test. The purpose of this was to both describe the cohort and to roughly estimate selection bias. The incidence rate for virological failure was calculated as the number of patients with a first VL ≥ 1000 copies over the total observation time. Using Cox proportional hazard regressions, we computed hazard ratios (HR) and respective 95% confidence intervals (CI). Significant variables from the bivariable analyses were investigated for confounding and effect modification using Mantel–Haenszel statistics and Woolf’s tests, as well as testing for co-linearity. Only dichotomized versions of variables were included in multivariable analyses. Variables were selected for multivariable analysis based on results from bivariable and stratified analyses. Where effect modification was identified in stratified analysis, we tested whether the addition of interaction terms significantly improved the model fit based on Akaike information criterion and analysis of variance. Only complete cases, thus without missing information for any of the variables selected in the univariate analysis, were considered for multivariable analysis. The final multivariable model was selected based on step-wise forward and backward Cox proportional hazards regression using the Akaike information criterion and likelihood ratio tests. The model proportional hazards assumption was tested using scaled Schoenfeld residuals. Assumptions of non-linearity was assessed visually. All analyses were two-tailed, with a significance level of 0.05, and carried out using R statistical software version 3.6.0 (Foundation for Statistical Computing, Vienna, Austria).

## Results

### Inclusion

**Fig. 2:**
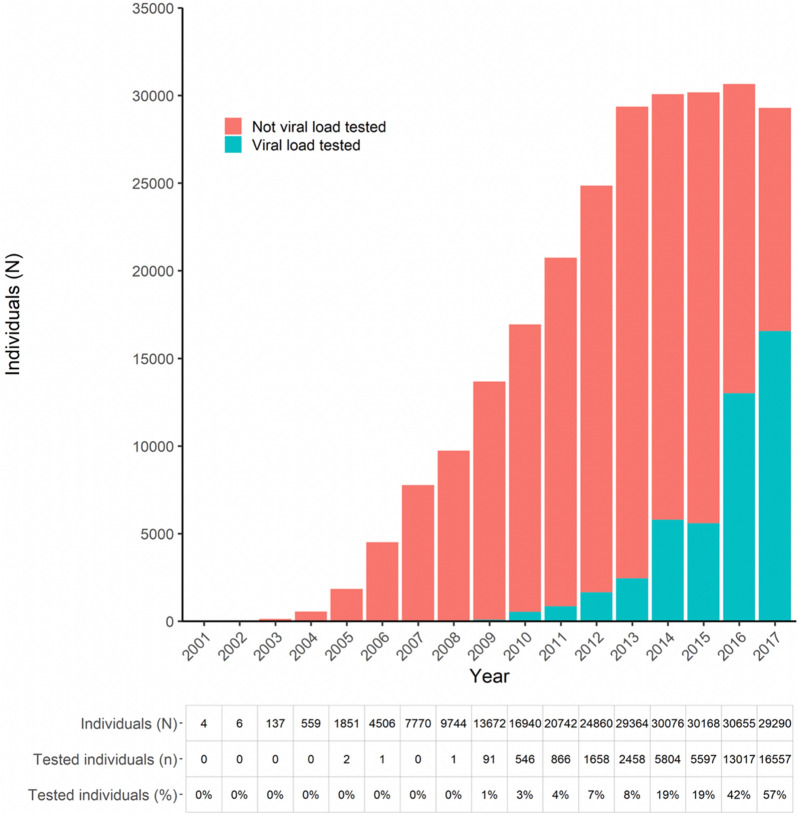
Number of people with at least one visit per year, categorized by receipt of at least one viral load test result in the same year

As illustrated in the Fig. 1, during the period 2003–2017 there were 51,010 patients enrolled in MSF programmes. 5,873 (11.5%) patients in the cohort were LFU or died before ART was initiated. Among patients who started on ART, 35,356 (78.3%) received > 6 months standard first line treatment. Among the 35,356, there were 7,858 (22.2%) who initiated treatment before 2009, before VL monitoring was implemented; and 27,498 (77.8%) initiating treatment during or after 2009. Among the 35,356, we recorded 140,779 person-years of follow-up time. During this time, 25,260 (71.4%) patients had at least one HIV VL test result available. HIV VL test coverage increased over time, with below 10% of individuals having a visit in 2013 and a VL test during the same year, to 57% in 2017 (Fig. 2).

Patients may have had multiple visits and multiple tests in one year however only the first result in a specific year was considered. A patient is included in the total count of each year they were receiving ART for at least part of the year.

### Baseline characteristics

Baseline characteristics of the first-line cohort, stratified by having received an HIV VL test, are presented in Table 1. Among 25,260 patients included in the analysis of virological outcomes, the median age was 33.1 years (IQR 28.0–39.1) and 54.7% were male. The median observation time was 5.4 years (IQR 3.7–7.9). The median CD4 count was 143 cells/mL (IQR 55–264) in 10,236 patients tested. Nearly half (45.6%) presented with WHO stage III or IV disease. Approximately one in four of this cohort (n = 6,656; 26.4%) were diagnosed with tuberculosis at baseline. Overall, 9,861 (39.0%) patients had one episode of low-level viremia and in 2,438 (24.7%) patients this occurred more than once. There were 2,440 (9.7%) patients LFU at least once, and 419 (1.66%) more than once. Most patients (86.7%, n = 21,918) were late at least once for a scheduled appointment. When considering delays to all scheduled appointments in total, about one in four (n = 6,005, 23.8%) had a cumulative appointment delay greater than 60 days.

**Table 1:** Baseline characteristics of the first-line cohort by receipt of HIV VL testing (n = 35,356)

Variable	Value	Total (n = 35,356)	(%)	HIV VL test result not available (n = 10,096)	(%)	HIV VL test result available (n = 25,260)	(%)	*P*-value*
Age at ART initiation > 19 years		32,387	91.6	9774	96.8	22,613	89.5	< 0.001
Gender (Female)		15,749	44.5	4294	42.5	11,455	45.3	0.02
Divorced		10	0.0	8	0.1	2	0.0	0.108
Married		20,165	57.0	6190	61.3	13,975	55.3	< 0.001
Separated		2178	6.2	673	6.7	1505	6.0	0.363
Single		8163	23.1	2010	19.9	6153	24.4	< 0.001
Widow		3927	11.1	1058	10.5	2869	11.4	0.409
Man who has sex with men		250	0.7	55	0.5	195	0.8	0.437
History of injection drug use		2785	7.9	1331	13.2	1454	5.8	< 0.001
History of sex work		508	1.4	120	1.2	388	1.5	0.37
History of blood transfusion		584	1.7	143	1.4	441	1.7	0.482
Economic migrant		675	1.9	241	2.4	434	1.7	0.053
History of imprisonment		515	1.5	169	1.7	346	1.4	0.498
Displaced person		106	0.3	41	0.4	65	0.3	0.434
Having HIV + partner		1902	5.4	760	7.5	1142	4.5	< 0.001
Baseline body mass index < 18.5 kg/m^2^		6829	19.3	1592	15.8	5237	20.7	< 0.001
	Missing	21,289	60.2	6091	60.3	15,198	60.2	
Baseline WHO stage	1	11,501	32.5	3426	33.9	8075	32.0	< 0.001
	2	1108	3.1	261	2.6	847	3.4	
	3	9710	27.5	2406	23.8	7304	28.9	
	4	5516	15.6	1336	13.2	4180	16.5	
	Missing	7521	21.3	2667	26.4	4854	19.2	
Baseline Tuberculosis		8754	24.8	2088	20.7	6666	26.4	< 0.001
Baseline CD4 (cells/mL)	< 200	8434	23.9	2013	19.9	6421	25.4	< 0.001
	200–500	4668	13.2	1,483	14.7	3185	12.6	
	> 500	928	2.6	298	3.0	630	2.5	
	Missing	21,326	60.3	6302	62.4	15,024	59.5	
Time on ART (years)	< 2	6773	19.2	4174	41.3	2599	10.3	< 0.001
	2 – 5	11,984	33.9	3500	34.7	8484	33.6	
	> 5	16,599	46.9	2422	24.0	14,177	56.1	
History of no treatment change		11,355	32.1	5411	53.6	5944	23.5	< 0.001
	Missing	204	0.6	117	1.2	87	0.3	
History of low viremia		9861	27.9	NA	NA	9861	39.0	
Frequency of low viremia	1	7423	21.0	NA	NA	7423	29.4	
	≥ 2	2438	6.9	NA	NA	2438	9.7	
History of lost-to-follow-up		3850	10.9	1410	14.0	2440	9.7	< 0.001
Number of times lost-to-follow-up	1	3176	9.0	1155	11.4	2021	8.0	< 0.001
	2	512	1.4	194	1.9	318	1.3	
	≥ 3	162	0.5	61	0.6	101	0.4	
Cumulative appointment delay ≥ 60 days		8852	25.0	2847	28.2	6005	23.8	< 0.001
Cumulative appointment delay (days)	1–59	21,507	60.8	5594	55.4	15,913	63.0	< 0.001
	60–181	4492	12.7	1286	12.7	3206	12.7	
	182–364	1709	4.8	586	5.8	1123	4.4	
	≥ 365	2651	7.5	975	9.7	1676	6.6	

*Chi2 test with Holm correction

Comparison between patients who did or did not have at least one HIV VL test showed that those who had HIV VL test results tended to be younger (median 33.1 years vs. 34.1 years; p < 0.001), had been on ART after 2009 for a longer time (median 5.4 years vs. 2.8 years, *p* < 0.001), and tended to have lower CD4 counts at ART initiation (median 143 cells/mL vs. 189 cells/mL; *p* < 0.001). Baseline tuberculosis was diagnosed more frequently among those who received HIV VL testing (24.8 vs. 20.7%; *p* < 0.001) and a lower proportion of them had a history of LFU (10.9 vs. 14.0%; *p* < 0.001). History of injecting drug use was less frequently reported among those with access to viral load testing (5.8 vs. 7.9%; *p* < 0.001). Those who had access to HIV VL testing had more episodes of late appointments, but a lower cumulative number of days late, and only 25% of them accumulated ≥ 60 days late for appointments, in comparison with 28.2% of those who had never received a HIV VL test (*p* < 0.001).

### HIV VL cascade

**Fig. 3:**
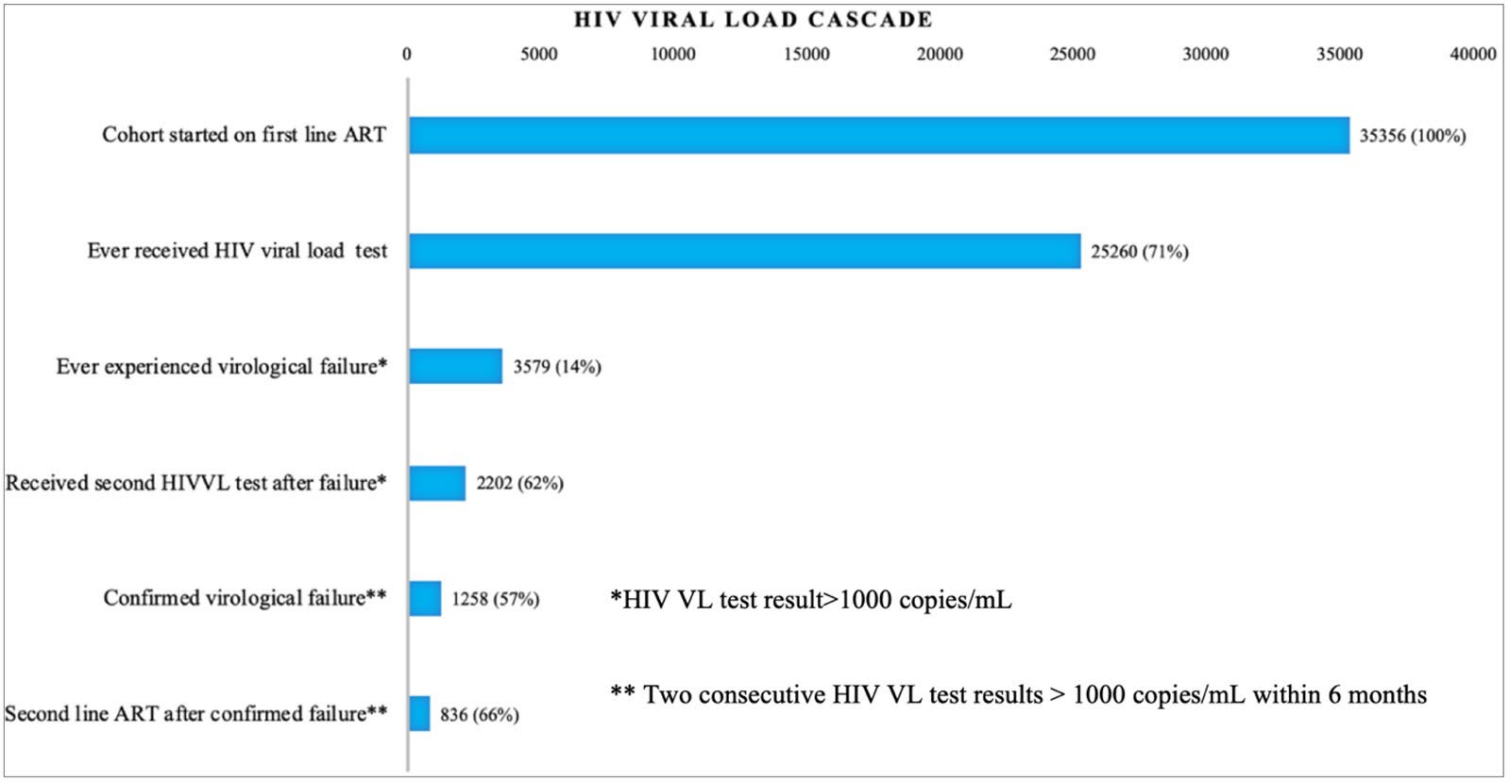
Viral load testing cascade among the first-line cohort (n = 35,356). The % against each bar are calculated using the total cohort number in the above bar as the denominator

Of 25,260 patients with at least one VL test result available, 3579 (14.2%) had documented virological failure, with a calculated incidence of failure of 2.5 per 100 person-years (3579 patients with failure during 143,160 years of follow-up). Among those with virological failure, 2202 (61.5%) had a consecutive VL test within six months of the first test that showed virological failure (Fig. 3). Of those with a consecutive VL test 1258 (57.1%) individuals had confirmed virological failure. Among those with confirmed virological failure, 836 (66.5%) switched to second-line ART within six months since confirmed virological failure. Among those with confirmed virological failure, the median time between the virological failure and confirmed virological failure was 3.6 months (IQR 2.4–4.8) and the median time between ART initiation and confirmed virological failure was 3.7 months (IQR 2.3–5.7).

### Predictors of virological failure

Using univariate regression, patients whose marital status was single (HR 1.66; 95% CI 1.54–1.78 *p* < 0.001), who were economic migrants (HR 1.63; 95% CI 1.30–2.05; *p* < 0.001), those with baseline BMI < 18.5 kg/m2 (HR 1.38; 95% CI 1.25–1.53; *p* < 0.001), CD4 > 500 cells/mL (HR 1.64; 95% CI 1.34–2.00; *p* < 0.001), or WHO stage two (vs. WHO stage one; HR 1.26; 95% CI 1.06–1.49; *p* < 0.001) were more likely to have virological failure (Table 2). Also PLHIV who experienced low-level viremia (HR 1.59; 95% CI 1.49–1.79; *p* < 0.001), were LFU at least once (HR 1.80; 95% CI 1.65–1.96; *p* < 0.001), or had a cumulative appointment delay over 60 days (vs. those who were never late; HR 1.69, 95% CI 1.58–1.81, *p* < 0.001) were more likely to experience virological failure. Females (HR 0.86; 95% CI 0.81–0.92; *p* < 0.001) or those with age > 19 years at ART initiation (0.36; 95% CI 0.35–0.42; *p* < 0.001) had lower hazards of virological failure.

**Table 2:** Crude hazard ratios (HR) for virological failure among PLHIV with more than 6 months of first-line ART and at least one VL test (n = 25,260)

Variable	No virological failure (n = 21,681)		Virological failure (n = 3,579)		HR (CI 95%)	*P* value*
	N	%	N	%		
Age at ART initiation > 19 years	19,752	87.3	2861	12.7	0.36 (0.35–0.42)	< 0.001
Female	9954	86.7	1501	13.3	0.86 (0.81–0.92)	< 0.001
Divorced	NA	NA	0		NA	
Married	12,295	88.0	1680	12.0	0.71(0.66–0.75)	< 0.001
Separated	1271	84.5	234	15.5	1.90 (0.95–1.23)	0.228
Single	4948	80.4	1205	19.6	1.66 (1.54–1.78)	< 0.001
Widow	2533	88.2	336	11.8	0.73 (0.65–0.81)	< 0.001
MSM	163	83.6	32	16.4	1.81 (0.77–1.54)	0.646
History of IDU	1260	86.7	194	13.3	1.13 (0.98–1.31)	0.099
History of sex work	319	82.2	69	17.8	1.24 (0.98–1.58)	0.074
History of transfusion	391	88.7	50	11.3	0.77 (0.58–1.02)	0.063
Economic migrant	357	82.3	77	17.7	1.63 (1.30–2.05)	< 0.001
History of imprisonment	304	87.9	42	12.1	1.02 (0.75–1.39)	0.889
History of displacement	56	86.2	9	13.8	1.27 (0.66–2.45)	0.472
Having HIV + partner	994	87.0	148	13.0	0.92 (0.78–1.08)	0.297
BMI < 18.5 kg/m3	4339	82.9	898	17.1	1.38 (1.25–1.53)	< 0.001
Baseline WHO stage						
1	6979	86.4	1096	13.6	Ref	
2	696	82.2	151	17.8	1.26 (1.06–1.49)	0.008
3	6263	85.7	1041	14.3	0.91 (0.84–0.99)	0.033
4	3602	86.2	578	13.8	0.83 (0.75–0.91)	< 0.001
Baseline CD4 > 500 cells/mL	530	84.1	100	15.9	1.64 (1.34–2.00)	< 0.001
Baseline Tuberculosis	5757	86.4	909	13.6	0.88 (0.81–0.95)	< 0.001
No ART regimen changes during observation time	4330	72.8	1664	27.2	6.60 (6.16–7.06)	< 0.001
History of low-level viremia	8032	81.5	1829	18.5	1.59 (1.49–1.70)	< 0.001
Frequency of low-level viremia						
0	13,649	88.6	1750	11.4	Ref	
1	6395	86.2	1028	13.8	1.15 (1.07–1.24)	< 0.001
≥ 2	1637	67.1	801	31.9	3.11 (2.86–3.38)	< 0.001
History of loss-to-follow-up	1837	75.3	603	24.7	1.80 (1.65–1.96)	< 0.001
Frequency of loss-to-follow-up						
0	19,844	87.0	2976	13.0	Ref	
1	1556	77.0	465	23.0	1.69 (1.53–1.86)	< 0.001
2	214	67.3	104	32.7	2.28 (1.87–2.77)	< 0.001
≥ 3	67	66.3	34	33.7	2.32 (1.66–3.26)	< 0.001
Cumulative appointment delay ≥ 60 days	4809	80.0	1196	20.0	1.69 (1.58–1.81)	< 0.001

*Wald test for the hazard ratio estimate of each exposure variable—comparing counts of those exposed with virological failure to those without

The multivariable analysis was conducted on 8,308 patients (32.9%) with complete information for all the variables required (Table 3), those presenting with baseline tuberculosis (aHR 1.39; 95% CI 1.14–1.49; *p* < 0.001), with a history of low-level viremia (aHR 1.60; 95% C I 1.42–1.81; *p* < 0.001), a history of LFU (aHR 1.24; 95% CI 1.41–1.52; p = 0.041), or being on the same treatment regimen since the start of treatment (aHR 1.37; 95% CI 1.07–1.76; *p* < 0.001) were associated with an increased hazard of failure, while controlling for other covariates. Starting ART at the age > 19 years was associated with 34% lower hazard of failure (95% CI 0.53–0.83; *p* < 0.001). Cumulative appointment delay was not significantly associated with failure after controlling for other covariates. We observed an interaction between sex work and gender (aHR 2.30; 95% CI 0.54–9.66; *p* = 0.26) and between gender and being single (aHR 1.43; 95% CI 1.08–1.89; *p* = 0.013). The differences between the characteristics of the population included in the final regression model and the entire population on first-line ART in this cohort, are presented in the Additional file 1: Table S1.

**Table 3:** Adjusted hazard ratios for virological failure among complete cases with more than 6 months of first-line ART and at least one VL test and complete data on key variables (n = 8,308)

Variable	aHR*	95% CI	*P*-value
Female	0.89	0.76–1.04	0.147
Age at ART initiation > 19 years	0.66	0.53–0.83	< 0.001
Baseline CD4 500 cells/mL	1.23	0.96–1.59	0.094
Baseline tuberculosis	1.30	1.14–1.49	< 0.001
Married	0.90	0.76–1.06	0.187
Single	0.83	0.66–1.03	0.089
History of sex work	0.71	0.18–2.86	0.633
History of IDU	1.05	0.77–1.43	0.764
History of loss-to-follow-up	1.24	1.01–1.52	0.041
History of low viremia	1.60	1.42–1.81	< 0.001
Cumulative appointment delay > = 60 days	0.94	0.79–1.12	0.473
No history of changing ART regimen	1.37	1.07–1.76	0.012

*Adjusted Hazard Ratio adjusted for variables presented in the table and history of ever receiving following categories of regimens: zidovudine/lamivudine/emtricitabine + abacavir/tenofovir;stavudine/zidovudine + lamivudine/emtricitabine + efavirenz; stavudine/zidovudine + lamivudine/emtricitabine + nevirapine; tenofovir/abacavir + lamivudine/emtricitabine + efavirenz or tenofovir/abacavir + lamivudine/emtricitabine + nevirapine; and time being on ART < or >  = 2 years since access to viral load monitoring 1 January 2009); interaction between sex work and gender and gender and being single included in the model

## Discussion

Our study investigated virological outcomes in PLHIV receiving first-line ART in Myanmar. During the long observation period, a total of 25, 260 (71.4%) PLHIV received at least one VL test and 14.2% experienced virological failure (2.5 per 100 person-years). Our results are comparable with previous reports from resource-limited settings, where virological failure occurred in 4.3–34.0% PLHIV on first-line ART [14, 15, 29–32]. Previous studies from Myanmar reported good long-term immunological and virological treatment outcomes among PLHIV on treatment [33], with one cohort experiencing a virological failure rate of 3.2 per 100 person-years [5]. In general, higher virological suppression rates have been reported in Asia than in Africa [34], although any comparison of virological outcomes is challenging, as local VL monitoring guidelines differ, and study follow-up times vary between the cohorts.

We showed that the risk of virological failure was lower among those initiating ART if they were aged > 19 years (90% of the study cohort) compared to those with or younger than 19 years. This finding is similar to that reported in other studies. The higher risk of failure among children and adolescents may be explained by suboptimal adherence, lack of paediatric drug formulations, and lack of care models responsive to the specific needs of these subgroups [5, 17, 35–37]. Patients in our cohort study, with baseline tuberculosis were at higher risk of virological failure, consistent with findings from other studies, which identified advanced HIV disease as a strong predictor of treatment failure [5, 14, 17, 38–40]. Almost 10% of our participants were LFU at least once, and the vast majority (86.8%) had been late for at least one appointment. This is in concordance with other studies showing temporary disengagement from care can be very common in these cohorts (11–77%) [21–25]. When LFU and appointment delay are not measured continuously, but only at a given moment in time, the frequency of treatment interruptions is very likely to be underestimated [21]. Our study relied on a rigorously updated programme database with regards to visit dates, which allowed us to identify delay and treatment interruptions. The cumulative appointment delay was < 60 days for 63% of the cohort. In the univariate analysis cumulative appointment delay ≥ 60 days was correlated with higher risk of virological failure (p < 0.001), but when controlling for other variables the association was not significant. However, having at least one single time point with treatment interruption of at least 60 days while being LFU was associated with increased risk of failure. This is similar to findings from other contexts [5, 39]. Adding cumulative appointment delay in the risk of virological failure analysis was not valuable in this cohort, but different cut-off values for cumulative appointment delay or delay per year on ART could be explored in future analyses.

A systematic review reported that history of treatment change was associated with an approximately 2.5-fold higher risk of virological failure in cohorts in Myanmar and Malawi [14]. In our cohort, PLHIV who remained on the same first-line ART regimen during the study period were at a higher risk of failure. It is possible that previous reports used a different definition of “treatment change”. In our cohort, patients with treatment changes may have been followed up more closely, with better management of adverse events and possibly a lower risk of drug-drug interactions.

Increasing evidence shows that low-level viremia is associated with unfavorable treatment outcomes. A large multicentre cohort in South Africa detected low-level viremia in 23% of PLHIV, with risk of subsequent failure in this group observed as 2.6 times higher (95% CI 2.5–2.6; *p* < 0.0001) than in PLHIV who did not experience low-level viremia [19]. In our study, 39% of patients had at least one episode of low-level viremia, and a history of low viremia was associated with an increased risk of treatment failure. A study from Sweden reported chronic low-level viremia in 31% of their population, with 2.1 times higher (95% CI 1.3–3.6) risk of mortality when compared with PLHIV without a history of low-level viremia [20].

There is an effective and life-saving second-line ART regimen, but delayed switch is particularly problematic in patients with advanced HIV disease. Current recommendations for the management of virological failure rely on a public health approach. Programmes in resource-limited settings use a threshold of ≥ 1000 copies/mL to identify failure and recommend switching to second-line ART when virological failure is confirmed in a second sample [41]. Some have argued that in settings with no access to drug resistance testing, such approaches might delay introduction of effective and life-saving second-line ART regimens and might increase risk of resistance accumulation, which in turn with further compromise effectiveness of second-line treatment; this would be particularly problematic in patients with advanced HIV disease and it has been argued that in some circumstances switching to second-line treatment could be considered in patients with a single VL showing viremia above 1000 copies/mL [42].

Guidelines on virological monitoring and the management of treatment failure have been changing over time [42–44]. In our study until 2016 most of our patients had a VL done based on immunological and/or clinical criteria. Only after 2012 did a threshold of VL ≥ 1000 copies/mL become an indication for switching to second-line ART. Nevertheless, in this study cohort since 2009 61.5% of patients with viremia ≥ 1000 copies/mL received a follow-up VL. Virological failure was confirmed among 57.1% of those with a follow-up VL, with 66.5% of the latter being switched to second-line ART. The implementation of VL monitoring in resource-limited settings is a challenge. A study from Swaziland reported an increase of follow-up VL coverage to 84% in recent years, however, the proportion of patients with confirmed virological failure switched to second-line ART remained low (43.2%) [45]. Similarly, in South Africa and Lesotho only 25–30% of patients in need were switched to second-line treatment in a timely manner [46, 47]. Even though enhanced adherence counselling has been reported as an effective strategy to identify those truly in need of second-line ART in settings with limited access to drug resistance testing, only 53.4% (95% CI 40.1–66.8%) of those who received such counselling and were identified as in need of second-line ART were switched, according to a systematic review from 2019 [48]. A previous study from Myanmar highlights the importance of timely switching to second-line ART, as one-third of those who did not switch died or were LFU from care [5].

In short, routine VL monitoring reduces mortality when used together with adherence support [48] and a timely switch to effective treatment [49, 50], but ensuring coverage of VL and second-line ART for those with a diagnosis of virological failure remains a huge challenge. When coverage is low, the overall benefit from VL scale-up might be lower than anticipated. To improve programme performance along the VL cascade, innovative approaches, such as “mHealth” [51] or “nurse-champions” [52] can be effective. Furthermore, it might be important to prioritize and differentiate VL testing in those at a higher risk of failure, in settings where barriers for scale-up exist. For instance, the management of PLHIV who re-engage in care after being LFU requires more frequent VL monitoring [26] and possibly a faster switch to second-line treatment, especially if they present with clinical signs of advanced HIV disease. Considering the correlation between low-level viremia and treatment failure [19, 53, 54] and mortality [20], the threshold of ≥ 1000 copies/mL for enrollment into enhanced adherence support and switching to second-line ART may need to be revised. A more differentiated approach to VL monitoring, guided by the increasing body of evidence on predictors of virological failure and mortality among patients with low-level viremia and/or virological failure, may result in better outcomes for those most at risk.

Our study evaluated a large cohort with a long study period. It used real-life programme data with complete data on appointment delays, including LFU. However, 28.6% of the cohort had no VL during the observation period and data for various baseline characteristics were incomplete, which resulted in only a part of our cohort being included in the final multivariable model. The resulting selection bias might lower the internal validity of our study results and reduce the generalizability of our study findings. The burden of virological failure was assessed by looking at the first episode of virological failure only, despite knowing that PLHIV transit from suppressed to unsuppressed state multiple times during their time on ART. This might underestimate the total burden of failure in a cohort and multistate analysis of virological outcomes would be more appropriate. We did not investigate the reason why patients did not access VL, were delayed or LFU, or why switching to second-line ART was delayed. Further research on these topics is needed.

## Conclusion

VL monitoring is an important tool to improve programme outcomes. Suboptimal viral load cascade in resource-limited settings hampers the full potential of VL monitoring and it reduces its cost-effectiveness. Our study observed higher rates of virological failure among children and adolescents, in PLHIV with tuberculosis co-infection and those with history of LFU or who remain on one treatment regimen. Those subgroups might need more frequent virological and more intensive clinical monitoring. Growing evidence on the risk factors for unfavourable virological and clinical outcomes, may suggest the refinement of a differentiated approach to VL monitoring in growing and aging HIV cohorts in resource-limited settings.

## Supplementary Information

**Additional file 1:** **Table S1.** Characteristics of patients included in multivariable analysis compared to the total population on first line ART.

## Data Availability

Data are available on request. MSF has a managed access system for data sharing that respects MSF’s legal and ethical obligations to its patients to collect, manage and protect their data responsibility. Ethical risks include, but are not limited to the nature of MSF operations and target populations being such that data collected often involves highly sensitive data. The dataset supporting the conclusions of this article is available are available on request in accordance with MSF’s data sharing policy (available at: http://fieldresearch.msf.org/msf/handle/10144/306501). Requests for access to data should be made to data.sharing@msf.org.

## Abbreviations

aHR: Adjusted hazard ratio
ART: Antiretroviral treatment
BMI: Body mass index
CI: Confidence intervals
HIV: Human Immunodeficiency Virus
IDU: Injection drug use
IQR: Interquartile range
LFU: Lost-to-follow-up
MSF: Médecins Sans Frontières
MSM: Man who has sex with men
PLHIV: People living with HIV
VL: Viral load
WHO: World Health Organization
