# Supplementary material for: Predictors of virological failure among people living with HIV receiving first line antiretroviral treatment in Myanmar: retrospective cohort analysis
Source: AIDS Res Ther. 2021 Apr 21;18:16. doi: 10.1186/s12981-021-00336-0 (PMC8059266; doi:10.1186/s12981-021-00336-0)
Supplement: Supplementary file 1 — Additional file 1: Table S1. Characteristics of patients included in multivariable analysis compared to the total population on first line ART. [file 12981_2021_336_MOESM1_ESM.docx]

**Table 1 (Additional File 1): Characteristics of patients included in multivariable analysis (n = 8,308) compared to the total population on first line ART (n = 35,356) for viral load**

| Variable | Value | Included in multivariable analysis  (n) | (%) | Not included in multivariable analysis  (n) | (%) | P-value |
| --- | --- | --- | --- | --- | --- | --- |
| Age at ART initiation >19 years |  | 7487 | 90.1 | 32387 | 91.6 | <0.001 |
| Gender (Female) |  | 3591 | 43.2 | 15749 | 44.5 | 0.031 |
| Divorced |  | 1 | 0.0 | 10 | 0.0 | 0.757 |
| Married |  | 4530 | 54.5 | 20165 | 57.0 | <0.001 |
| Separated |  | 513 | 6.2 | 2178 | 6.2 | 1 |
| Single |  | 2101 | 25.3 | 8163 | 23.1 | <0.001 |
| Widow |  | 906 | 10.9 | 3927 | 11.1 | 1 |
| Man who has sex with men |  | 74 | 0.9 | 250 | 0.7 | 0.092 |
| History of injection drug use |  | 391 | 4.7 | 2785 | 7.9 | <0.001 |
| History of sex work |  | 169 | 2.0 | 508 | 1.4 | <0.001 |
| Economic migrant |  | 161 | 1.9 | 675 | 1.9 | 1 |
| History of imprisonment |  | 135 | 1.6 | 515 | 1.5 | 0.401 |
| Baseline body mass index <18.5kg/m ^2^ |  | 1879 | 22.6 | 6829 | 19.3 | <0.001 |
|  | Missing | 4714 | 56.7 | 21289 | 60.2 |  |
| WHO stage at ART initiation | 1 | 3247 | 39.1 | 11501 | 32.5 | <0.001 |
|  | 2 | 327 | 3.9 | 1108 | 3.1 |  |
|  | 3 | 2958 | 35.6 | 9710 | 27.5 |  |
|  | 4 | 1776 | 21.4 | 5516 | 15.6 |  |
|  | Missing | 0 | 0.0 | 7521 | 21.3 |  |
| Baseline Tuberculosis |  | 2994 | 36.0 | 8754 | 24.8 | <0.001 |
| Baseline CD4 (cells/mL) | <200 | 5262 | 63.3 | 8434 | 23.9 | <0.001 |
|  | 200-500 | 2580 | 31.1 | 4668 | 13.2 |  |
|  | >500 | 466 | 5.6 | 928 | 2.6 |  |
|  | Missing | 0 | 0.0 | 21326 | 60.3 |  |
| History of low viremia |  | 3498 | 42.1 | 9861 | 27.9 | <0.001 |
| Frequency of low viremia | 1 | 2640 | 31.8 | 7423 | 21.0 | <0.001 |
|  | ≥2 | 858 | 10.3 | 2438 | 6.9 |  |
| History of no treatment change |  | 1922 | 23.1 | 11355 | 32.1 | <0.001 |
| Time on ART (years) | <2 | 931 | 11.2 | 6773 | 19.2 | <0.001 |
|  | 2-5 | 2368 | 28.5 | 11984 | 33.9 |  |
|  | >5 | 5009 | 60.3 | 16599 | 46.9 |  |
| History of lost-to-follow up |  | 760 | 9.1 | 3850 | 10.9 | <0.001 |
| Frequency of lost-to-follow up | 1 | 629 | 7.6 | 3176 | 9.0 | <0.001 |
|  | 2 | 102 | 1.2 | 512 | 1.4 |  |
|  | ≥3 | 29 | 0.3 | 162 | 0.5 |  |
| Cumulative appointment delay >=60 days |  | 1936 | 23.3 | 8852 | 25.0 | <0.001 |
| Cumulative appointment delay (days) | 1 - 59 | 5194 | 62.5 | 21507 | 60.8 | 0.03 |
|  | 60 - 181 | 996 | 12.0 | 4492 | 12.7 |  |
|  | 182-364 | 375 | 4.5 | 1709 | 4.8 |  |
|  | ≥365 | 565 | 6.8 | 2651 | 7.5 |  |
